# The association between *CTSZ* methylation in peripheral blood and breast cancer in Chinese women

**DOI:** 10.3389/fonc.2023.1148635

**Published:** 2023-05-18

**Authors:** Jinyu Li, Xiajie Zhou, Lixi Li, Longtao Ji, Jiaqi Li, Yunhui Qu, Zhi Wang, Yutong Zhao, Jie Zhang, Feifei Liang, Jingjing Liu, Wanjian Gu, Rongxi Yang, Fei Ma, Liping Dai

**Affiliations:** ^1^ School of Basic Medical Sciences & The First Affiliated Hospital, Zhengzhou University, Zhengzhou, Henan, China; ^2^ Department of Epidemiology and Biostatistics, School of Public Health, Nanjing Medical University, Nanjing, Jiangsu, China; ^3^ Department of Medical Oncology, National Cancer Center, National Clinical Research Center for Cancer, Cancer Hospital, Chinese Academy of Medical Sciences & Peking Union Medical College, Beijing, China; ^4^ BGI College, Zhengzhou University, Zhengzhou, Henan, China; ^5^ Henan Institute of Medical and Pharmaceutical Sciences & Henan Key Medical Laboratory of Tumor Molecular Biomarkers, Zhengzhou University, Zhengzhou, Henan, China; ^6^ Department of Clinical Laboratory in the First Affiliated Hospital & Key Clinical Laboratory of Henan Province, Zhengzhou University, Zhengzhou, Henan, China; ^7^ Department of Clinical Laboratory, Jiangsu Province Hospital of Chinese Medicine, Nanjing, Jiangsu, China

**Keywords:** *CTSZ*, DNA methylation, breast cancer, case-control study, Chinese women

## Abstract

**Purpose:**

Previous studies have shown that DNA methylation in peripheral blood may be associated with breast cancer (BC). To explore the association between the methylation level of the Cathepsin Z *(CTSZ)* gene in peripheral blood and BC, we conducted a case–control study in the Chinese population.

**Methods:**

Peripheral blood samples were collected from 567 BC cases, 635 healthy controls, and 303 benign breast disease (BBD) cases. DNA extraction and bisulfite-specific PCR amplification were performed for all samples. The methylation levels of seven sites of the *CTSZ* gene were quantitatively determined by Mass spectrometry. The odds ratios (ORs) of CpG sites were evaluated for BC risk using per 10% reduction and quartiles analyses by logistic regression.

**Results:**

Our analysis showed that five out of the seven CpG sites exhibited significant associations with hypomethylation of *CTSZ* and BC, compared to healthy controls. The highest OR was for Q2 of *CTSZ*_CpG_1 (OR: 1.62, *P*=0.006), particularly for early-stage breast cancer in the case of per 10% reduction of *CTSZ*_CpG_1 (OR: 1.20, *P*=0.003). We also found that per 10% reduction of *CTSZ*_CpG_5 (OR: 1.39, *P*=0.004) and CTSZ_CpG_7,8 (OR: 1.35, *P*=0.005) were associated with increased BC risk. Our study also revealed that four out of seven CpG sites were linked to increased BC risk in women under 50 years of age, compared to healthy controls. The highest OR was for per 10% reduction of *CTSZ*_CpG_1 (OR: 1.47, *P*<0.001). Additionally, we found that BC exhibited lower methylation levels than BBD at *CTSZ*_CpG_4 (OR for Q1: 2.18, *P*<0.001) and *CTSZ*_CpG_7,8 (OR for Q1: 2.01, *P*=0.001). Furthermore, we observed a correlation between methylation levels and tumor stage, ER, and HER2 status in BC patients.

**Conclusion:**

Overall, our findings suggest that altered *CTSZ* methylation levels in peripheral blood may be associated with breast cancer, particularly in young women, and may serve as a potential biomarker for early-stage BC.

## 1 Introduction

In 2020, Global cancer statistics reported that the leading cause of disability and mortality among women worldwide is breast cancer (BC) (1). As early as 2018, the global cancer statistics showed that BC accounts for 15% of all types of female cancer deaths (2). BC is a complex and heterogeneous disease. Previous research has suggested there is a significant correlation between the incidence of BC and race and ethnicity, which is also normally associated with a range of risk factors including exposure to hormones, diet, and lifestyles (3). Despite the advance in the prevention and treatment that can improve the survival rates for BC patients, there is still a lack of effective early diagnosis methods (4). One study demonstrated that early detection of breast cancer could reduce overall mortality by 37% (5). Recently, a large body of compelling evidence indicates that the potential of epigenetic modifications for the prevention, diagnosis, and treatments of BC has an important impact on the development and progression (6).

Epigenetics refers to changes in gene expression levels that are not based on changes in gene sequence. Epigenetics includes DNA methylation, chromatin remodeling, histone modifications, and non-coding RNA, which can produce heritable variations without changing the DNA sequence (7). DNA methylation is a covalent modification. DNA methylation occurs exclusively on cytosine nucleotides and almost always in the context of CpG. As an important epigenetic mechanism, DNA methylation usually refers to the transfer of methyl groups to the C5 position of the cytosine to form a 5-methylcytosine under the action of DNA methyltransferase (DNMT). 5-methylcytosine, the methylated form of DNA base cytosine, is the main epigenetic marker in mammalian nuclear DNA and may be involved in gene transcription regulation, posttranscriptional modification to affect the maturation, stability, and translation of mRNA molecules. It is also one of the indispensable epigenetic adaptations that affect cell proliferation, apoptosis, differentiation, cell cycle, and transformation (8). The transcriptional start sites of many genes encoding tumor suppressors, such as retinoblastoma-associated protein 1 (RB1), MLH1, p16 and BRCA1, among others, lie within CGIs (CpG islands). In somatic cells, gene body methylation is a major cause of cancer gene mutations in tumor suppressor genes. In general, the tumor genome is hypomethylated, yet the potential role of methylation of CpG-poor promoters, enhancers, insulators, repetitive elements and gene bodies in cancer is almost completely unknown (9). With regards to tissue samples, hypermethylation in the promoter regions of tumor suppressor genes often leads to transcriptional repression and decreased gene expression (10, 11). However, genome-wide DNA hypomethylation may promote tumor formation by increasing chromosomal instability. Therefore, the identification of methylation changes in DNA from peripheral blood and their association with breast cancer have been of great interest for early disease diagnosis and treatment (12–14).

Cathepsin Z (*CTSZ*) is a differentiated member of the cathepsins. *CTSZ* has only mono- and dipeptidyl carboxypeptidase activity and being responsible for cleavage. *CTSZ* also activates some molecules, such as the integrin called lymphocyte function associated antigen (LFA-1), the focal adhesion kinase (FAK), the SRC kinase, and others. *CTSZ* is expressed in several primary tumor types, such as PCa (prostate cancer), colorectal, gastric, liver, melanoma, and pancreatic neuroendocrine tumors (15). In some of these studies, *CTSZ* was found in tumor-associated macrophages. It indicated that this protease played a role in the antitumor immune response (16). So far, there have been no studies about the correlation between *CTSZ* methylation and breast cancer. Thus, we hypothesized that methylation level on the site of *CTSZ* may effect breast cancer. It has been reported that DNA methylation from tissue or circulating blood tumor DNA may be a promising biomarker and could predicted and distinguished different subtypes of BC (17). To provide evidence for the selection of early markers for breast cancer, we explored the methylation levels between the *CTSZ* gene and breast cancer among Chinese women through a case-control design in this study.

## 2 Materials and methods

### 2.1 Study populations

This study was approved by the ethics committees of Nanjing Medical University, the Chinese Academy of Medical Sciences and the First Affiliated hospital of Zhengzhou University. The written informed consent was obtained from each recruited participants before the peripheral blood samples collection. All the individuals were Chinese women. BC was confirmed by postoperative pathological analyses in each case.

In this study, we collected 1505 whole blood samples, all from peripheral blood. 567 BC patients with the mean age of 50.5 years old (range: 22.0-85.0 years old) were from the Cancer Hospital of the Chinese Academy of Medical Sciences and the First Affiliated Hospital of Zhengzhou University. The criteria for breast cancer patients were those who had been diagnosed with cancer for the first time without any treatment. A total of 635 healthy female control group were enrolled by the Health Center in Nanjing Hospital of Chinese Medicine. The mean age was 47.5 years old (range: 24.0-94.0 years old). The physical examination report was normal, no tumor history, no family history of tumor disease. In addition, a total of 303 patients with BBD (Benign breast disease) were collected from the Cancer Hospital of the Chinese Academy of Medical Sciences and the First Affiliated Hospital of Zhengzhou University. The mean age was 48.0 years old (range: 24.0-78.0 years old). The criteria for the recruitment of BBD were women who had two or more biopsies within six months and the results were benign and no tumor history. Additional clinical characteristics information is described in detail in **Table 1**.

**Table 1:** Clinical characteristics of BC cases.

Sample Characteristics	Type	BC Cases (N=567)	
		N	%
Age	≤50 years	280	49.38
	>50 years	287	50.62
Tumor subtype	Luminal A	156	27.51
	Luminal B	189	33.33
	Triple-negative	53	9.35
	Others	133	23.46
	Unknow	36	6.35
Tumor size	T0	15	2.65
	T1	165	29.10
	T2	259	45.68
	T3	43	7.58
	T4	18	3.17
	Unknow	57	11.82
Tumor stage	Stage 0	15	2.65
	Stage I	96	16.93
	Stage II	224	39.51
	StageIII	110	19.40
	StageIV	29	5.11
	Unknow	93	16.40

### 2.2 Sample collecting and processing

Peripheral blood was collected using Ethylene Diamine Tetraacetic Acid (EDTA) tubes and stored at −80°C. Whole-genome DNA was extracted from the whole blood sample according to the instructions of the DNA extraction kit (TANTICA, China). The above-described extracted DNA was treated with bisulfite using the EZ-96 DNA Methylation Gold Kit (Zymo Research, USA) kit according to the above instructions.

### 2.3 MALDI-TOF mass spectrometry

**Figure 1:**
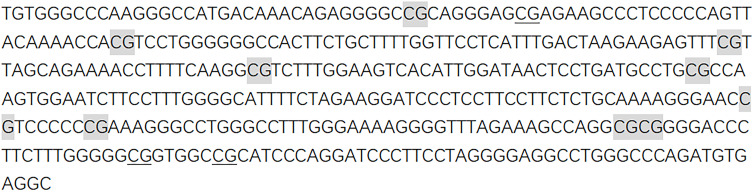
Sequences of *CTSZ* amplicon for MassARRAY methylation analysis. The sequence of the *CTSZ* amplicon examined by the EpiTyper assay. The EpiTyper assay determined the methylation levels of 12 CpGs in this amplicon, and yielded 7 distinguishable peaks. The 9 measurable CpG sites are highlighted, and the undetectable CpG sites are underlined.

Agena matrix-assisted laser desorption/ionization (MALDI)- time-of-flight (TOF) mass spectrometry (Agena Bioscience, San Diego, California, United States) described by Yang et al. was used to determine the levels of DNA methylation semiquantitatively (18, 19). In short, the bisulfite-converted DNA was amplified by bisulfite-specific primers (**Figure 1**).

Forward primer: aggaagagagTGTGGGTTTAAGGGTTATGATAAAT, reverse primer: cagtaatacgactcactatagggagaaggctACCTCACATCTAAACCCAAACCT. Upper case letters represent specific regions of the sequence, while lower case letters are used for non-specific tags. Neither the primers nor the amplicons were overlapped with any known SNPs. Most of the mass peaks detected by this method have only one CpG site and a few contain two. For example, *CTSZ*_CpG_7 and *CTSZ*_CpG_8 after the EpiTyper treatment are located in the same fragment, and thus the mass peak shows the average methylation level of *CTSZ*_CpG_7 and *CTSZ*_CpG_8 (expressed as *CTSZ*_CpG_7,8 in the manuscript). This is applied to *CTSZ*_CpG_9,10 as well. The polymerase chain reaction (PCR) products were processed according to the standard protocol of Agena EpiTyper Assay. Then, it is cleaned with resin and dispensed to 384 SpectroCHIP *via* Nanodispenser. MassARRAY system was used for data collection and the EpiTYPER v1.2 software was used for calculation. In all the processing and analyses, BC cases and controls were handled in parallel.

### 2.4 Statistical analyses

SPSS version 26.0 was used for all the statistical analysis. Age and batche adjusted logistic regression was used to assess the association between BC risk and the methylation levels of *CTSZ*, as well as between BC and BBD cases. The odds ratios (ORs) with 95% confidence interval (CI) of CpG sites were evaluated for BC risk using per 10% reduction and quartiles analyses by logistic regression. OR>1 indicates that *CTSZ* methylation level is a risk factor for BC. Tests for linear trend across quartiles of methylation levels of each CpG site in *CTSZ* were conducted using the logistic regression model. The group with the highest methylation level (Q4) was used as the reference group, and the other three groups were set as dummy variables to enter the regression model. The signifcant trend indicated that the relative risk of breast cancer showed a rising trend as the methylation level decreased.The interquartile analysis was performed by calculating the quartiles of methylation levels at each site in BC case and controls. We used Kruskal–Wallis test and Mann–Whitney U test to analyze the significant correlation between *CTSZ* methylation level and the different clinical characteristics. *P <*0.05 was considered statistical significance, it indicates that there is a correlation between two groups. All statistical tests were two-sided.

## 3 Results

### 3.1 Association between hypomethylation of *CTSZ* in peripheral blood and BC

**Figure 2:**
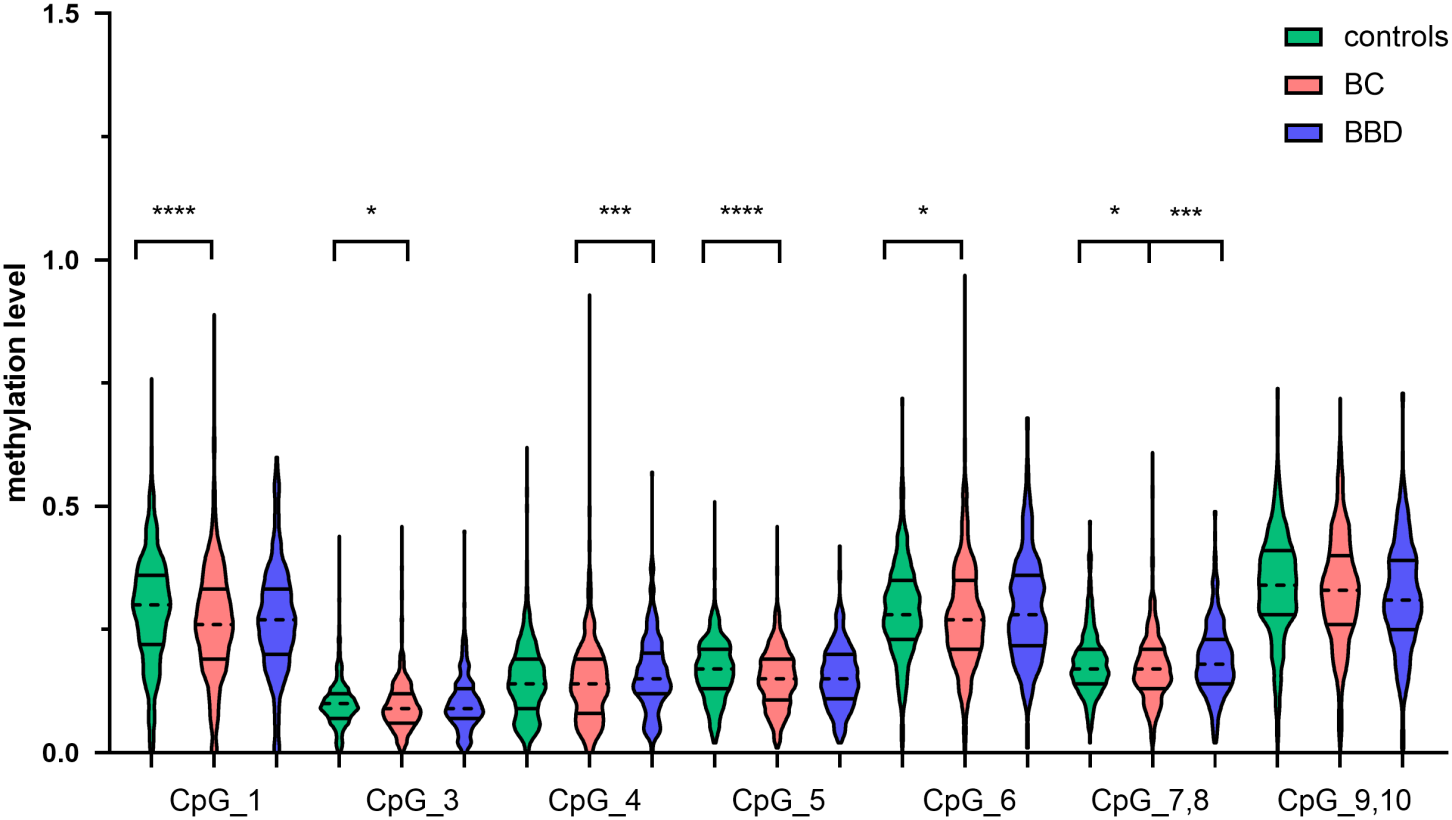
The *CTSZ* methylation levels of BC, BBD and controls on each site and their correlation. DNA extraction and bisulfite-specific PCR amplification were performed for 567 BC cases, 303 BBD cases and 635 controls. The methylation levels of seven sites of the *CTSZ* gene were quantitatively determined by Mass spectrometry. Mann-Whitney U test was used to analyze the methylation levels (median±IQR) between two groups. *P<0.05, ***P<0.001, ****P<0.0001.

To investigate the association between *CTSZ* methylation and BC, a case-control study with 567 BC cases and 635 age matched control groups was conducted. As shown in **Figure 2**, there was a significant association between BC and controls on the site of *CTSZ*_CpG_1, *CTSZ*_CpG_3, *CTSZ*_CpG_5, *CTSZ*_CpG_6 and *CTSZ*_CpG_7,8. Meanwhile, BC had lower methylation level compared to controls (**Table 2**). Interquartile analyses were also performed to assess the ORs of methylation levels in all the *CTSZ* CpG sites to the risk of BC. Comparing to the highest methylation level (Q4), *CTSZ*_CpG_1, *CTSZ*_CpG_3, *CTSZ*_CpG_5 *CTSZ*_CpG_6 and *CTSZ*_CpG_7,8 showed significant association between hypomethylation of *CTSZ* and BC compared to controls with the highest OR for Q1 of *CTSZ*_CpG_5 (OR:1.646, *P*=0.013, **Table 3**). The effects of methylation in *CTSZ*_CpG_1, *CTSZ*_CpG_3, *CTSZ*_CpG_5 and *CTSZ*_CpG_6 were also enhanced along with lower interquartiles (*P*_trend_ for *CTSZ*_CpG_1<0.001, *P*_trend_ for *CTSZ*_CpG_3 = 0.035, *P*_trend_ for *CTSZ*_CpG_5 < 0.001, *P*_trend_ for *CTSZ*_CpG_6 = 0.010). In addition, a significant result was found at *CTSZ*_CpG_7,8 when comparing Q1 and Q4(OR=1.434, 95%CI:1.031-1.996, *P*=0.032), but no significant *P*_trend_ existed. Although we did not find a correlation between *CTSZ*_CpG_9,10 and BC, we did find that OR tended to increase as the interquartile decrease (*P*_trend_ for *CTSZ*_CpG_9,10 = 0.041; **Table 3**).

**Table 2:** Methylation differences of *CTSZ* between BC cases, controls and BBD.

CpG Sites	BC Cases (N=567) Median (IQR)	Controls(N=635) Median (IQR)	BBD(N=303) Median (IQR)	*P*-value^a^	*P-*value^b^
*CTSZ*_CpG_1	0.26 (0.19-0.33)	0.30 (0.22-0.36)	0.27 (0.20-0.34)	<0.001*	0.455
*CTSZ*_CpG_3	0.09 (0.06-0.12)	0.10 (0.07-0.12)	0.09 (0.07-0.13)	0.033*	0.335
*CTSZ*_CpG_4	0.14 (0.08-0.19)	0.14 (0.09-0.19)	0.15 (0.12-0.21)	0.396	<0.001*
*CTSZ*_CpG_5	0.15 (0.11-0.19)	0.17 (0.13-0.21)	0.15 (0.11-0.20)	<0.001*	0.431
*CTSZ*_CpG_6	0.27 (0.21-0.35)	0.28 (0.23-0.35)	0.28 (0.21-0.36)	0.011*	0.089
*CTSZ*_CpG_7.8	0.17 (0.13-0.21)	0.17 (0.14-0.21)	0.18 (0.14-0.23)	0.019*	<0.001*
*CTSZ*_CpG_9.10	0.33 (0.26-0.40)	0.34 (0.28-0.41)	0.31 (0.25-0.39)	0.073	0.110

a Mann-Whitney U-test of BC and controls comparison.

b Mann-Whitney U-test of BC and BBD comparison.

*Statistically significant.

**Table 3:** Inter-quartile analysis for *CTSZ* methylation level between BC cases and controls.

CpG sites	Quartiles (methylation range)	Controls N (%)	BC cases N (%)	Total	OR (95%CI)	*P*-value	*P*trend
*CTSZ*_CpG_1	Q4 (> 0.33)	224(35.3)	141(24.9)	365	1	–	<0.001 *
	Q3 (≤ 0.33)	173(27.2)	139(24.6)	312	1.211(0.880-1.665)	0.239	
	Q2 (≤ 0.26)	118(18.6)	143(25.3)	261	1.618(1.145-2.287)	0.006 *	
	Q1 (≤ 0.19)	120(18.9)	143(25.2)	263	1.370(0.933-2.011)	0.108	
*CTSZ*_CpG_3	Q4 (> 0.12)	137(21.6)	131(23.1)	268	1	–	0.035 *
	Q3 (≤ 0.12)	182(28.7)	118(20.8)	300	1.484(1.062-2.075)	0.021 *	
	Q2 (≤ 0.09)	150(23.6)	117(20.7)	267	0.794(0.564-1.118)	0.187	
	Q1 (≤ 0.07)	166(26.1)	200(35.4)	366	1.171(0.850-1.612)	0.334	
*CTSZ*_CpG_4	Q4 (> 0.19)	144(22.7)	126(22.3)	270	1	–	0.365
	Q3 (≤ 0.19)	163(25.7)	134(23.7)	297	0.927(0.657-1.308)	0.667	
	Q2 (≤ 0.14)	161(25.4)	140(24.7)	301	0.828(0.578-1.186)	0.303	
	Q1 (≤ 0.09)	167(26.2)	166(29.3)	333	0.904(0.634-1.288)	0.575	
*CTSZ*_CpG_5	Q4 (> 0.19)	207(32.6)	124(21.9)	331	1	–	<0.001 *
	Q3 (≤ 0.19)	164(25.8)	139(24.6)	303	1.266(0.907-1.766)	0.165	
	Q2 (≤ 0.15)	137(21.6)	137(24.2)	274	1.362(0.959-1.935)	0.084	
	Q1 (≤ 0.11)	127(20.0)	166(29.3)	293	1.646(1.110-2.441)	0.013 *	
*CTSZ*_CpG_6	Q4 (> 0.35)	146(22.9)	129(22.8)	275	1	–	0.010 *
	Q3 (≤ 0.35)	170(26.8)	110(19.4)	280	0.784(0.543-1.133)	0.196	
	Q2 (≤ 0.28)	172(27.1)	145(25.6)	317	0.995(0.676-1.465)	0.981	
	Q1 (≤ 0.22)	147(23.2)	182(32.2)	329	1.478(1.066-2.049)	0.019 *	
*CTSZ*_CpG_7.8	Q4 (> 0.21)	149(23.5)	126(22.3)	275	1	–	0.091
	Q3 (≤ 0.21)	164(25.8)	125(22.1)	289	0.983(0.692-1.397)	0.845	
	Q2 (≤ 0.17)	141(22.2)	125(22.1)	266	1.082(0.743-1.575)	0.211	
	Q1 (≤ 0.14)	181(28.5)	190(33.5)	371	1.434(1.031-1.996)	0.032 *	
*CTSZ*_CpG_9.10	Q4 (> 0.40)	159(25.0)	134(23.7)	293	1	–	0.041 *
	Q3 (≤ 0.40)	173(27.2)	130(22.9)	303	0.881(0.627-1.238)	0.592	
	Q2 (≤ 0.33)	157(24.7)	135(23.9)	292	0.980(0.688-1.397)	0.841	
	Q1 (≤ 0.27)	146(23.1)	167(29.5)	313	1.128(0.780-1.631)	0.112	

^*^Statistically significant

### 3.2 The methylation differences of *CTSZ* between BC and BBD

To evaluate the methylation differences of *CTSZ* between BC and BBD. We conducted a case-control study, which had 567 BC cases and 303 age matched BBD cases. As shown in **Figure 2**, there was a significant association between BC and BBD on the sites of *CTSZ*_CpG_4 and *CTSZ*_CpG_7,8. We found a significant association between hypomethylation of *CTSZ* and BC compared to BBD (**Table 2**). However, we further carried out an interquartile analysis, we found that BC had lower methylation level compared to BBD on the site of *CTSZ*_CpG_4 (OR(95%CI) for Q1 of *CTSZ*_CpG_4: 2.179 (1.445-3.278), *P*<0.001) and *CTSZ*_CpG_7,8 (OR (95%CI) for Q1 of *CTSZ*_CpG_7,8: 2.012 (1.329-2.874), *P*=0.001, **Table 4**). Moreover, with the lower interquartile of methylation levels, the higher risk of BC on site of *CTSZ*_CpG_4, *CTSZ*_CpG_7,8 (*P*_trend_ for *CTSZ*_CpG_4 = 0.001, *P*_trend_ for *CTSZ*_CpG_5 = 0.002) were be found.

**Table 4:** Inter-quartile analysis for *CTSZ* methylation level between BC cases and BBD.

CpG sites	Quartiles (methylation range)	BBD N (%)	BC cases N (%)	Total	OR (95%CI)	*P*-value	*P*trend
*CTSZ*_CpG_1	Q4 (> 0.33)	75 (24.8)	141 (24.9)	172	1	–	0.601
	Q3 (≤ 0.33)	62 (20.5)	110 (19.5)	216	0.875 (0591-1.665)	0.870	
	Q2 (≤ 0.27)	88 (29.2)	152 (26.8)	240	1.076 (0.731-1.584)	0.711	
	Q1 (≤ 0.20)	77 (25.5)	163 (28.8)	240	1.036 (0.679-1.580)	0.504	
*CTSZ*_CpG_3	Q4 (> 0.12)	79 (26.2)	131 (23.2)	210	1	–	0.313
	Q3 (≤ 0.12)	64 (21.2)	118 (20.8)	182	0.900 (0.593-1.364)	0.618	
	Q2 (≤ 0.09)	86 (28.5)	171 (30.2)	257	0.873 (0.595-1.282)	0.489	
	Q1 (≤ 0.06)	73 (24.1)	146 (25.8)	219	0.868 (0.582-1.295)	0.488	
*CTSZ*_CpG_4	Q4 (> 0.19)	91 (30.2)	126 (22.3)	217	1	**-**	0.001 *
	Q3 (≤ 0.19)	72 (23.8)	134 (23.7)	206	1.344 (0.905-1.996)	0.143	
	Q2 (≤ 0.14)	84 (27.8)	140 (24.7)	224	1.171 (1.797-1.721)	0.423	
	Q1 (≤ 0.09)	55 (18.2)	166 (29.3)	221	2.179 (1.445-3.278)	<0.001 *	
*CTSZ*_CpG_5	Q4 (> 0.19)	80 (26.5)	124 (21.9)	204	1	–	0.305
	Q3 (≤ 0.19)	68 (22.5)	139 (24.6)	207	0.761 (0.507-1.142)	0.187	
	Q2 (≤ 0.15)	69 (22.8)	137 (24.2)	206	0.804 (0.536-1.207)	0.293	
	Q1 (≤ 0.11)	85 (28.2)	166 (29.3)	251	0.827 (0.562-1.218)	0.337	
*CTSZ*_CpG_6	Q4 (> 0.35)	78 (25.8)	129 (22.8)	207	1	–	0.304
	Q3 (≤ 0.35)	75 (24.8)	141 (24.9)	216	0.867 (0.581-1.293)	0.484	
	Q2 (≤ 0.27)	74 (24.6)	143 (25.3)	217	0.834 (0.559-1.245)	0.376	
	Q1 (≤ 0.21)	75 (24.8)	153 (27.0)	228	0.792 (0.532-1.178)	0.249	
*CTSZ*_CpG_7.8	Q4 (> 0.22)	83 (27.5)	103 (18.2)	186	1	–	0.002 *
	Q3 (≤ 0.22)	81 (26.8)	148 (26.1)	229	1.510 (1.013-2.252)	0.043 *	
	Q2 (≤ 0.17)	72 (23.8)	163 (28.8)	235	1.972 (1.314-2.959)	0.001 *	
	Q1 (≤ 0.13)	66 (21.9)	152 (26.9)	218	2.012 (1.329-2.874)	0.001 *	
*CTSZ*_CpG_9.10	Q4 (> 0.40)	61 (20.2)	134 (23.8)	195	1	–	0.093
	Q3 (≤ 0.40)	75 (24.8)	157 (27.7)	232	1.085 (0.718-1.638)	0.699	
	Q2 (≤ 0.32)	77 (25.5)	128 (22.6)	205	1.360 (0.896-2.065)	0.148	
	Q1 (≤ 0.26)	89 (29.5)	147 (25.9)	236	1.348 (0.900-2.019)	0.147	

^*^Statistically significant.

### 3.3 Association between *CTSZ* methylation and BC by age

Because age plays an important role in BC risk, we calculated the association between BC and methylation levels by age (20). Here, we stratified the subjects by the age of 50 years. Then we conducted interaction analysis between *CTSZ* methylation and age, and found that there were significant difference on site of *CTSZ*_CpG_1, *CTSZ*_CpG_5, *CTSZ*_CpG_6, *CTSZ*_CpG_7,8. In the group of under the age of 50 (including 50 years), there were 279 BC cases and 392 controls. We conducted a binary logistic regression analysis and controlled for experimental batches, we found that hypomethylation of *CTSZ*_CpG_1, *CTSZ*_CpG_5, *CTSZ*_CpG_6, *CTSZ*_CpG_7,8 was associated with BC, while the highest OR for per 10% reduction of *CTSZ*_CpG_1 was 1.47(95%CI:1.19-1.82, *P*<0.001, **Table 5**). In the contrast, in the group of age>50 including 289 BC cases and 243 controls, there was none of the CpG sites significantly correlated with BC risk.

**Table 5:** Age-Stratified Association Between *CTSZ* Methylation and BC.

CpG Sites	BC Cases Median (IQR)	Controls Median (IQR)	OR (95%CI)^c^ per-10% Methylation	*P*-value^c^
≤50 years old (279BC cases vs 392 controls)				
*CTSZ*_CpG_1	0.26 (0.19-0.33)	0.31 (0.24-0.37)	1.47 (1.19-1.82)	<0.001^*^
*CTSZ*_CpG_5	0.15 (0.11-0.19)	0.18 (0.13-0.21)	1.47 (1.12-1.92)	0.006^*^
*CTSZ*_CpG_6	0.26 (0.20-0.33)	0.29 (0.23-0.34)	1.19 (1.01-1.41)	0.038^*^
*CTSZ*_CpG_7.8	0.16 (0.13-0.20)	0.17 (0.14-0.21)	1.39 (1.06-1.85)	0.013^*^
>50 years old (289 BC cases vs 243 controls)				
*CTSZ*_CpG_1	0.26 (0.20-0.35)	0.28 (0.19-0.35)	1.10 (0.86-1.39)	0.456
*CTSZ*_CpG_5	0.14 (0.10-0.19)	0.15 (0.11-0.20)	1.38 (0.98-1.96)	0.063
*CTSZ*_CpG_6	0.27 (0.22-0.36)	0.28 (0.22-0.36)	1.03 (0.78-1.29)	0.842
*CTSZ*_CpG_7.8	0.17 (0.14-0.22)	0.18 (0.14-0.23)	1.28 (0.88-1.55)	0.186

c Logistic regression, adjusted for experimental batches.

^*^ Statistically significant.

### 3.4 Association between *CTSZ* methylation levels and BC at early stage

To calculate the association between methylation levels and BC at early stage (stage0-stageII), we conducted a binary logistic regression analysis in a case-control study, which had 335 BC cases and 635 age matched controls. When adjusted for age and experimental batches, there was a significant association between hypomethylation of *CTSZ* and BC cases compared to controls for per 10% reduction of *CTSZ*_CpG_1 (OR:1.20; *P*=0.003); *CTSZ*_CpG_5 (OR:1.39; *P*=0.004); *CTSZ*_CpG_7,8 (OR:1.35; *P*=0.005) in **Table 6**. Meanwhile, we further carried out an interquartile analyses and found that there were association between BC and CTSZ methylation on the site of *CTSZ*_CpG_1, *CTSZ*_CpG_5 and *CTSZ*_CpG_7,8 where the highest OR of *CTSZ*_CpG_5 was 1.815(95%CI:1.217-2.707, *P*=0.003, **Table 7**). Moreover, with the lower interquartile of methylation levels, the higher risk of BC on site of *CTSZ*_CpG_1, *CTSZ*_CpG_3, *CTSZ*_CpG_5, *CTSZ*_CpG_7,8 were found (*P*_trend_ for *CTSZ*_CpG_1<0.001, *P*_trend_ for *CTSZ*_CpG_3 = 0.010, *P*_trend_ for *CTSZ*_CpG_5 < 0.001, *P*_trend_ for *CTSZ*_CpG_7,8 = 0.045; **Table 7**).

**Table 6:** Methylation differences of *CTSZ* between controls and BC cases at early stage.

CpG Sites	BC Cases (N=335) Median (IQR)	Controls (N=635) Median (IQR)	OR (95%CI)^c^ per-10% Methylation	*P*-value^c^
*CTSZ*_CpG_1	0.27 (0.19-0.34)	0.30 (0.22-0.36)	1.20 (1.06-1.35)	0.003^*^
*CTSZ*_CpG_3	0.09 (0.06-0.12)	0.10 (0.07-0.12)	0.85 (0.65-1.11)	0.234
*CTSZ*_CpG_4	0.15 (0.09-0.20)	0.14 (0.09-0.19)	1.06 (0.89-1.26)	0.493
*CTSZ*_CpG_5	0.15 (0.10-0.19)	0.17 (0.13-0.21)	1.39 (1.11-1.75)	0.004^*^
*CTSZ*_CpG_6	0.27 (0.21-0.36)	0.28 (0.23-0.35)	0.91 (0.79-1.03)	0.139
*CTSZ*_CpG_7.8	0.17 (0.13-0.21)	0.17 (0.14-0.21)	1.35 (1.09-1.67)	0.005^*^
*CTSZ*_CpG_9.10	0.33 (0.27-0.40)	0.34 (0.28-0.41)	0.98 (0.87-1.11)	0.768

c Logistic regression, adjusted for age and experimental batches.

*Statistically significant.

**Table 7:** Inter-quartile analysis for *CTSZ* methylation level between BC cases and controls at early stage.

CpG sites	Quartiles (methylation range)	BC cases N (%)	controls N (%)	Total	OR (95%CI)	*P*-value	*P*trend
*CTSZ*_CpG_1	Q4 (> 0.36)	63 (18.8)	152 (23.9)	215	1	–	<0.001*
	Q3 (≤ 0.36)	66 (19.7)	175 (26.6)	241	0.913 (0.604-1.380)	0.667	
	Q2 (≤ 0.29)	105 (31.3)	165 (25.9)	270	1.503 (1.022-2.211)	0.038*	
	Q1 (≤ 0.21)	101 (30.2)	143 (22.5)	244	1.526 (1.027-2.265)	0.036*	
*CTSZ*_CpG_3	Q4 (> 0.12)	73 (21.8)	137 (21.6)	210	1	–	0.010*
	Q3 (≤ 0.12)	64 (19.1)	182 (28.7)	246	0.699 (0.461-1.058)	0.090	
	Q2 (≤ 0.09)	75 (22.4)	150 (23.6)	225	0.968 (0.643-1.455)	0.874	
	Q1 (≤ 0.07)	123 (36.7)	166 (26.1)	289	1.272 (0.875-1.849)	0.208	
*CTSZ*_CpG_4	Q4 (> 0.19)	88 (26.3)	144 (22.7)	232	1	–	0.402
	Q3 (≤ 0.19)	80 (23.9)	163 (25.7)	243	0.841 (0.574-1.232)	0.375	
	Q2 (≤ 0.14)	82 (24.5)	161 (25.4)	243	0.795 (0.543-1.163)	0.237	
	Q1 (≤ 0.09)	85 (25.3)	167 (26.2)	252	0.821 (0.562-1.199)	0.308	
*CTSZ*_CpG_5	Q4 (> 0.20)	59 (17.6)	175 (27.6)	234	1	–	<0.001*
	Q3 (≤ 0.20)	74 (22.1)	152 (23.9)	226	1.449 (0.963-2.179)	0.075	
	Q2 (≤ 0.16)	93 (27.7)	155 (24.4)	248	1.687 (1.137-2.504)	0.009*	
	Q1 (≤ 0.12)	109 (32.6)	153 (24.1)	262	1.815 (1.217-2.707)	0.003*	
*CTSZ*_CpG_6	Q4 (> 0.35)	85 (22.9)	146 (22.8)	231	1	–	0.075
	Q3 (≤ 0.35)	61 (26.8)	170 (19.4)	231	0.685 (0.457-1.027)	0.067	
	Q2 (≤ 0.28)	79 (27.1)	172 (25.6)	251	0.859 (0.584-1.263)	0.439	
	Q1 (≤ 0.22)	110 (23.2)	147 (32.2)	257	1.315 (0.909-1.901)	0.146	
*CTSZ*_CpG_7.8	Q4 (> 0.21)	75 (25.4)	149 (23.5)	224	1	–	0.045*
	Q3 (≤ 0.21)	69 (20.6)	164 (25.8)	233	0.980 (0.650-1.478)	0.924	
	Q2 (≤ 0.17)	68 (20.3)	141 (22.2)	209	1.174 (0.769-1.793)	0.458	
	Q1 (≤ 0.14)	123 (36.7)	181 (28.5)	304	1.579 (1.078-2.312)	0.019*	
*CTSZ*_CpG_9.10	Q4 (> 0.40)	78 (23.3)	159 (25.0)	237	1	–	0.131
	Q3 (≤ 0.40)	67 (20.0)	152 (23.9)	219	0.946 (0.634-1.410)	0.784	
	Q2 (≤ 0.34)	84 (25.1)	150 (23.6)	234	1.156 (0.787-1.697)	0.459	
	Q1 (≤ 0.28)	106 (31.6)	174 (27.4)	280	1.165 (0.804-1.688)	0.419	

*Statistically significant.

### 3.5 *CTSZ* methylation levels and the clinical characteristics of BC patients

The relationship between *CTSZ* methylation level and the clinical characteristics of BC patients was investigated in 567 BC cases. Patients with higher tumor stage had lower methylation levels in *CTSZ*_CpG_4 (*P*<0.05, **Table 8**). Meanwhile, the negative status of ER was correlated with lower methylation levels at *CTSZ*_CpG_7,8 (*P*=0.028) while the hypermethylation level at *CTSZ*_CpG_7,8 was related to the HER2 positive status(*P*=0.020). However, the other six sites did not show this trend. In addition, our results showed no correlation of methylation level of *CTSZ* with tumor size, tumor subtype and the level of Ki67 (**Table 8**).

**Table 8:** The association between *CTSZ* methylation level and the clinical characteristics of BC patients.

Clinical characteristics	Group (N)	Median of age	Median of methylation levels in the CpG sites of *CTSZ*						
			*CTSZ*_CpG_1	*CTSZ*_CpG_3	*CTSZ*_CpG_4	*CTSZ*_CpG_5	*CTSZ*_CpG_6	*CTSZ*_CpG_7.8	*CTSZ*_CpG_9.10
Tumor stage (474)	Stage 0&I (111)	51.0	0.27	0.08	0.13	0.15	0.27	0.17	0.33
	Stage II (224)	51.5	0.27	0.09	0.13	0.15	0.27	0.16	0.33
	Stage III&IV (139)	50.0	0.26	0.10	0.12	0.15	0.25	0.16	0.33
	*P*-value (Kruskal-Wallis test)	0.375	0.677	0.407	0.010*	0.984	0.351	0.551	0.727
Tumor size (500)	Tis&T1 (181)	51.0	0.25	0.08	0.12	0.15	0.25	0.16	0.33
	T2 (259)	52.0	0.27	0.09	0.14	0.15	0.27	0.17	0.33
	T3&T4 (61)	48.0	0.27	0.09	0.14	0.14	0.25	0.16	0.34
	*P*-value (Kruskal-Wallis test)	0.135	0.636	0.500	0.647	0.527	0.359	0.894	0.967
Tumor subtype (531)	Luminal A (156)	48.0	0.27	0.09	0.14	0.16	0.26	0.16	0.33
	Luminal B (189)	51.0	0.26	0.09	0.14	0.15	0.28	0.17	0.32
	Triple-negative (53)	52.0	0.26	0.09	0.12	0.14	0.27	0.16	0.34
	Others (133)	52.0	0.26	0.08	0.14	0.15	0.26	0.16	0.34
	*P*-value (Kruskal-Wallis test)	0.628	0.424	0.879	0.806	0.678	0.266	0.168	0.898
Ki67^d^(535)	Low (129)	51.0	0.27	0.09	0.15	0.15	0.28	0.18	0.34
	High (406)	50.0	0.26	0.09	0.14	0.15	0.27	0.17	0.33
	*P*-value (Mann-Whitney U)	0.479	0.174	0.255	0.586	0.134	0.237	0.086	0.553
ER status (536)	ER negative (168)	49.0	0.24	0.09	0.13	0.14	0.26	0.16	0.32
	ER positive (368)	50.0	0.27	0.09	0.14	0.15	0.28	0.17	0.33
	*P*-value (Mann-Whitney U)	0.673	0.058	0.217	0.167	0.188	0.076	0.028*	0.886
PR status (545)	PR negative (219)	51.0	0.24	0.09	0.14	0.14	0.26	0.16	0.32
	PR positive (326)	50.0	0.27	0.09	0.14	0.15	0.28	0.17	0.33
	*P*-value (Mann-Whitney U)	0.389	0.055	0.384	0.630	0.113	0.085	0.081	0.415
HER2 status (528)	HER2 negative (207)	51.0	0.27	0.09	0.14	0.15	0.27	0.16	0.34
	HER2 positive (321)	50.0	0.26	0.09	0.14	0.14	0.28	0.17	0.32
	*P*-value (Mann-Whitney U)	0.859	0.137	0.669	0.384	0.214	0.627	0.020*	0.143

d Individuals with ≥ 20% and < 20% of Ki67 were considered as populations at high and low cell proliferation respectively. Compared their methylation level by non-parametric test.

*Statistically significant.

## 4 Discussion

Immune cells, such as monocytes, macrophages, and dendritic cells regularly express *CTSZ* (16). According to the above, we found a negative correlation between *CTSZ* methylation and BC in whole blood with more than 1500 subjects. In our study, four out of seven CpG sites showed significant correlation between BC and controls where BC cases had lower methylation level compared to controls in all sites. Case–control studies concluded that such genomic hypomethylation continuum can be evident at blood DNA level and may identify women at high-risk of BC (21). Some retrospective case–control studies also reported that breast cancer patients have lower global methylation levels compared to control groups in blood DNA (22). In conclusion, our results are consistent with those previous studies. Although there are no reports about the association between BC and BBD, it is worth doing some research. In our study, we found that there was a significant correlation between BC and BBD cases in the two out of seven CpG sites, meanwhile, BC cases had lower methylation than BBD cases in the six out of seven sites. Therefore, the methylation level of *CTSZ* in BBD cases is a potential indicator to assess the risk for BC. It can also be used as a reference to distinguish benign and malignant breast nodules.

Aging is a natural and irreversible process in human life. To date, it is clear that epigenetic changes, particularly DNA methylation, are associated with the aging process (23). Prospective studies reported that DNA methylation may serve as a measure of aging because of its strong association with age (24). In our study, we not only found that hypomethylation was associated with BC by age, but also validated the correlation between BC and methylation levels was younger than 50 years. Meanwhile, we found that BC cases had lower methylation levels compared to the control groups in each site. The mean age and menopause for Asian women is about 49 years old, and perimenopause refers to 3 to 4 years before menopause, which is 45-49 years old (25). Since estrogen levels decrease with age in Chinese women, we divided the age of the subjects into 50 years. Age is an important factor in alteration of DNA methylation levels (26). However, there have been rarely reports on the correlation between *CTSZ* methylation levels and age to date. According to the above, we inferred that *CTSZ* methylation levels in women who have breast cancer and younger than 50 (mostly premenopausal) may be related to age.

We further analyzed the blood-based *CTSZ* methylation and the clinical characteristics in 567 BC cases and 635 controls. The available clinical features of our study included tumor stage, tumor size, tumor subtype, Ki67 levels, estrogen receptor (ER) status, progesterone receptor (PR) status and human epidermal growth factor receptor 2 (HER2) status. We found patients with higher tumor stage had lower levels of methylation. This suggested that the methylation levels of *CTSZ* may be a means to assess early BC. In addition, we also found an association between ER status, HER2 status and BC on *CTSZ*_CpG_7,8 sites. Previous study have found that demethylated state of several matrix metalloproteinase promoter regions in tumors is associated with low expression of HER2 (27). In our study, *CTSZ* methylation was also significantly lower in HER2 negative BC cases. ER negative also had lower methylation levels compared to ER positive in BC cases. We propose that the expression of HER2 and ER status may influence methylation levels of *CTSZ*. In previous studies, HER2 positive BC has been associated with a higher incidence of DNA methylation in several genes, including PGR (which codes for PR), HSD17B4 (28). Alternatively, HER2 negative BC was associated with reduced methylation of PGR and HSD17B4. Our findings suggested that aberrant methylation of *CTSZ* in blood might be an important predictor of BC development and might be a biomarker for the prognosis of BC. Thus, we can hypothesize that there might be a significant association between *CTSZ* methylation and BC in younger women with higher level of estrogen and HER2 positive BC carriers.

## 5 Conclusion

Our study found associations between altered methylation and breast cancer in a large sample size of Chinese women. Specifically, we discovered that hypomethylation of the *CTSZ* gene is associated with early-stage breast cancer in premenopausal Chinese women. This finding may lead to the identification of potential biomarkers, such as CTSZ methylation, which could help improve early detection and treatment of breast cancer. We also identified a correlation between *CTSZ* methylation level in BC and tumor stage, ER status and HER2 status. Nevertheless, our findings can only suggest the blood-based DNA methylation markers as an early diagnostic marker for BC due to the limitations of the retrospective study. To determine whether these biomarkers could be constituted as early diagnostic markers for BC, prospective multicenter clinical studies with population-based cohorts should be mandatory for future studies.

## Data availability statement

The original contributions presented in the study are included in the article/supplementary materials. Further inquiries can be directed to the corresponding authors.

## Ethics statement

The studies involving human participants were reviewed and approved by Nanjing Medical University Chinese Academy of Medical Sciences Zhengzhou University. The patients/participants provided their written informed consent to participate in this study.

## Author contributions

RY, FM, and LD contributed to the study conception and design. XZ, LL, WG, JQL, JYL, JJL, and JZ performed material preparation. Data collection was performed by JYL, ZW, JJL, and YZ, and JQL and YZ performed analysis. The first draft of the manuscript was written by JYL and all authors commented on previous versions. All authors contributed to the article and approved the submitted version.
